# Core Competencies for Maritime Business Educators in the Digital Era

**DOI:** 10.3389/fpsyg.2022.915980

**Published:** 2022-07-12

**Authors:** Kum Fai Yuen, Lovevin Tan, Hui Shan Loh

**Affiliations:** ^1^School of Civil and Environmental Engineering, Nanyang Technological University, Singapore, Singapore; ^2^School of Business, Singapore University of Social Sciences, Singapore, Singapore

**Keywords:** competencies, maritime business education, digitalization, exploratory factor analysis, teaching performance

## Abstract

In light of digitalization, the objectives of this study are to (1) identify the emerging, core competencies of maritime business educators (MBE) and (2) examine their effects on perceived teaching performance. A systematic review of the contemporary literature was first performed to build a competency framework. Subsequently, an online survey was administered to 196 faculty members of the top 10 leading maritime universities as identified from the Worldwide Maritime School Rankings produced by Hong Kong Polytechnic University. The collected data were analyzed using Exploratory Factor Analysis to extract the main competencies of MBE. The results uncovered five key competencies from 22 sub-competencies. Thereafter, Hierarchical Regression Modeling was used to examine the effects of the key competencies on perceived teaching performance. After controlling for teaching experience and job position, it was found that the five competency requirements, in descending order of their importance, have significant positive effects on teaching performance: Pedagogy, Maritime, Interpersonal, Business and Digital. Academically, this study consolidates the literature and identifies the emerging core competencies that are expected from MBE in the digital era. The study also implicates education policy formulation, offering directions for institutions to allocate their resources, develop suitable training and assessment programs, and facilitate benchmarking.

## Introduction

Through digitalization, the maritime industry business models have transformed from a traditionally largely unskilled, labor-intensive industry to a capital-intensive, sophisticated one (Lau and Ng, 2015). This increases the need for highly trained individuals with sufficient skills to perform maritime specific business-related roles. Traditionally, higher education institutions offer maritime business courses to provide learners with the relevant skills and prepare them for jobs in the maritime sectors (Chen et al., 2018). The courses offered by these institutions enable maritime business graduates to gain working opportunities in the commercial side of marine operations, chartering, logistics, freight forwarding, supply chain, ship brokering and maritime law (MSC, 2020). The maritime business educators (MBE) play an important role in bridging the gap to supply industry-ready graduates. The competencies that they possess are associated with learners’ performance and achievement (Hill and Chin, 2018; Kolandan et al., 2020). Faculty members with extensive knowledge or experience in the Maritime, Logistics and Transportation industries are sought after to hold positions as MBE in higher level institutions. They are required to draw on their expertise and experience of the industry and impart them to learners. However, in a digital era, the knowledge and skills required by these educators are evolving rapidly, and may become outdated by the time the learners enter the workforce (Alop, 2019; Boulougouris et al., 2019). In order to develop a workforce that meets the present and future market demands, it is vital to ensure these educators possess relevant and up-to-date competencies. The concept of competencies can be interpreted in several ways, with connotations such as “competency,” “competencies” (Fisher, 2006). In the context of this research, it refers to a combination of practical and cognitive skills, knowledge, experience, attributes and behavior to conduct work successfully in accordance with standards, rules and procedures (Eraut, 1998; Guasch et al., 2010).

The literature on maritime education and workforce skills can be categorized into two research streams: seafarer education and training; and onshore maritime workforce skills. The first research stream, which is also the most extensively studied, focuses on seafaring education and training for on-board competencies. For instance, the International Convention on Standards of Training, Certification and Watchkeeping for Seafarers (STCW) sets the requirements for training of seafarers, and implementing of course curriculum in accordance with these requirements were discussed (Alfultis, 2019; Demirel, 2020). As the maritime industry undergoes digital transformation, it brings about challenges and opportunities for maritime education and training. Specifically, the introduction of unmanned ships will impact seafaring education whereby new skills and competencies are required to operate these ships (Ahvenjärvi, 2017; Senčila and Kalvaitienë, 2020). Both Alop (2019) and Johnson (2020) highlighted the imperative of curricular re-structuring in maritime education and training to catch up with the technology-driven innovations in the maritime industry. On the other hand, the rise of digitalization presents an opportunity for maritime education and training educators to integrate immersive technologies such as virtual reality and augmented reality into training programs, to reduce the gap between classroom-based training and real life (Mallam et al., 2019).

The second stream of research relates to onshore maritime workforce skills, which could be further divided into two categories: supply chain professionals and maritime business professionals. The research in the supply chain realm is fragmented in terms of the competency requirements of these professionals, with little agreement among researchers on the type of skills, categories and their terminologies (Tatham et al., 2017). According to Mageto and Luke (2020), six supply chain skills frameworks have received attention in the literature, including the business, logistics and management framework (Murphy and Poist, 1991), T-shaped framework (Mangan and Christopher, 2005), expertise level (Wu, 2006), supply chain management skills (Rahman and Qing, 2014), hard and soft skills (Abreu and Alcântara, 2015), and competence-performance framework (Lorentz et al., 2013). In view of the contemporary skills requirements of the supply chain industry, other researchers have advanced the existing frameworks. For instance, Mageto and Luke (2020) revised the business, logistics and management framework and reduced the number of skills from 83 to 36, while adding three ethics and environmental skills. With regards to maritime business workforce, some studies explored the knowledge and skills required by shipping professionals in their countries (Fernando et al., 2013; Han and Li, 2015). For example, Fernando et al. (2013) suggested that attaining skills such as analytical thinking and time management allow shipping professionals in Sri Lankan to achieve greater success. In addition, researchers that identified the employment requirements of maritime business graduates recognized the importance of communication and knowledge (Chen et al., 2017, 2018).

Despite the extant literature in maritime education, little research has explored the competency requirements of MBE. The main gaps in the maritime education are the lack of a holistic identification of competencies required by MBE and the examination of the competencies on teaching performance. In view of these aforementioned gaps in the literature, the first objective of this study is to provide a comprehensive identification of the competencies of MBE. This proposed framework consists of five key competencies and 22 sub-competencies for MBE. The five key competencies are (1) Business, (2) Pedagogy, (3) Interpersonal, (4) Digital, and (5) Maritime Foundational. The second objective is to examine the effects of the five key dimensions on teaching performance. The findings can be used by MBE to conduct self-assessment and identify their skills gap. At the institution level, the results can facilitate lifelong learning and course benchmarking, as it is crucial for MBE to deepen their skills to meet existing and emerging demands of the maritime sector (SkillsFuture, 2020a).

## Literature Review

**FIGURE 1:**
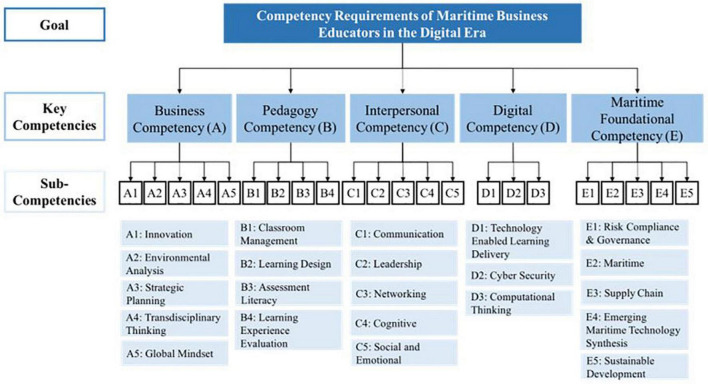
The MBE framework.

This section aims to identify the five key competencies of MBE, including Business, Pedagogy, Interpersonal, Digital and Maritime Foundational. These five key competencies and 22 sub-competencies are illustrated in a three-level hierarchical model as shown in Figure 1. The description and supporting references of each sub-competency are later illustrated in Table 1.

**TABLE 1:** Scale development.

Key competencies	Sub-competencies	Description	Sources
Business	Innovation	Apply creative thinking and foster a culture of innovation	Zhu et al., 2013; SkillsFuture, 2020b,c
	Environmental Analysis	Analyze global environmental landscape and respond to changes in competency demands	Alfultis, 2019; Peschl et al., 2020
	Strategic Planning	Assess organizational objectives to aid decision making in allocation of firms’ resources	Kotler and Murphy, 1981; Van Dam et al., 2010; Thai and Yeo, 2015; Hill and Chin, 2018
	Transdisciplinary Thinking	Apply concepts from multiple disciplines to guide decisions	Choi and Pak, 2006; Levinson, 2016; Chen et al., 2018; Alfultis, 2019
	Global Mindset	Recognize different cultural values and cultivate global perspective	Progoulaki and Roe, 2011; Goodwin, 2020
Pedagogy	Classroom Management	Establish a productive and stimulating learning environment	Doyle, 1980; Guerriero, 2014
	Learning Design	Analyze learner demographics to formulate delivery approaches that engage learners and fulfill learning outcomes	Mandinach and Gummer, 2013; Newble and Cannon, 2013; Bound and Chia, 2020
	Assessment Literacy	Gain clarity of purpose in assessment design and implement assessment tools to evaluate learner progress	Woytek, 2005; Lloyd, 2013; Ministry of Education, 2020; SkillsFuture, 2020b
	Learning Experience Evaluation	Measure effectiveness of learning materials through learners’ feedback and performance	Goe et al., 2008; SkillsFuture, 2020b
Interpersonal	Communication	Ability to convey message clearly, concisely and effectively	Parker, 2002; Blašková et al., 2014; Thai and Yeo, 2015; Chen et al., 2018; Serrano, 2019; Laar et al., 2020
	Leadership	Influence learner’s motivation through guidance and foster independent learning	Thai and Yeo, 2015; Tang and Sampson, 2018; SkillsFuture, 2020a,b
	Networking	Identify and establish relationships with stakeholders	SkillsFuture, 2020a,b
	Cognitive	Reasoning ability and general intelligence	Schmidt, 2002; Rotherham and Mead, 2003; Chamorro-Premuzic and Furnham, 2010; Bardach and Klassen, 2020
	Social and Emotional	Recognize and manage own emotions and those of others	Goleman et al., 2013; Sutton et al., 2015; Grant, 2017; Media, 2017
Digital	Technology Enabled Learning Delivery	Leverage electronic media and latest digital technologies for lesson deliveries	Allen and Seaman, 2014; Boulougouris et al., 2019; Simonson et al., 2019; Ministry of Education, 2020; Redecker, 2020; SkillsFuture, 2020b
	Cyber Security	Basic understanding of measures and techniques to protect integrity of data	Fireman, 2018; Alfultis, 2019; IMDA, 2020
	Computational Thinking	Reasoning and problem-solving skill	Wing, 2006; Sheldon, 2017; SkillsFuture, 2020b
Maritime Foundational	Risk Compliance and Governance	Understanding of laws and regulations of the industry	Murphy and Poist, 1991; Mangan and Christopher, 2005; Ziarati et al., 2010; Thai and Yeo, 2015; Boulougouris et al., 2019; Mageto and Luke, 2020
	Maritime-specific	Understanding concepts and industry practices that facilitate international trade from a maritime viewpoint	Shulman, 1986; Thai and Yeo, 2015; Chen et al., 2018; Ulferts, 2019
	Supply chain-specific	Understanding concepts and industry practices in supply chain discipline	Tatham et al., 2017; Mageto and Luke, 2020
	Emerging Technology Synthesis	Identify new and emerging technology in industry to support the learning materials	Chen et al., 2018; SkillsFuture, 2020b
	Sustainable Development	Understanding sustainable concepts and industry practices, including corporate social responsibility, sustainable shipping and green logistics	Abukhader and Jönson, 2004; Song and Panayides, 2012; EMSA, 2020; SkillsFuture, 2020a

Several studies have discussed the measurement of teaching performance of educators’ techniques and strategies. Teaching performance may be evaluated through students’ opinions (Rahimi et al., 2012), educators’ perception (Kaldi, 2009), graduate employability (Yorke and Knight, 2004; Yorke, 2006; Lee and Johnes, 2021) and assessment of learners’ acquired knowledge (Ahmed et al., 2015; Al-Jubari et al., 2019). It has also been found that educators are evaluated based on their research skills in addition to extensive professional experience as their research skills can enhance the classroom experience as well (Romero and Bobkina, 2021; Suyo-Vega et al., 2022). Among the factors that contribute positively to teaching effectiveness are pedagogy competency and knowledge (Goe et al., 2008), previous teaching performance and academic achievement scores (Corcoran and O’Flaherty, 2018). However, with parts of the learning set in virtual environments today, it is meaningful to investigate the relationships between key competencies of MBE and teaching performance.

### Business Competency

Business competency has become a fundamental aspect in every competencies framework for professionals in the education, maritime and supply chain industry (Van Dam et al., 2010; Thai, 2012; Chen et al., 2018; SkillsFuture, 2020a). In this age of rapid advancements, educators serve more than producers of knowledge, and they are expected to act as entrepreneurs themselves (Johnson, 2004). Business acumen is crucial to MBE to better analyze the environment and adapt quickly to new developments (SkillsFuture, 2020c). Therefore, business competency suggests five factors that would facilitate MBE’s dual role as a maritime business professional and educator: innovation, environmental analysis, strategic planning, transdisciplinary thinking and global mindset.

*Innovation* represents MBE’s ability to apply creative thinking and introduce new ideas that respond to both the internal and external environment. Creative thinking is viewed as an emerging critical skill as educators are required to adopt fresh perspectives to combine ideas and create improvements or solutions (SkillsFuture, 2020c). It is unrealistic to expect several generations of students to benefit from the same teaching approach and content (Zhu et al., 2013). Therefore, creative thinking would facilitate MBE to “think-out-of-the-box,” which is positively associated with learning design formulation. Furthermore, understanding the concept of creative thinking and demonstrating it would allow MBE to cultivate a culture of creativity for learners (SkillsFuture, 2020b).

*Environmental Analysis* is the capacity of MBE to analyze the global environmental landscape and respond to changes in the competency demands of the maritime education industry. A continuous process of scanning of the environment allows maritime professionals to better anticipate and react to change (Alfultis, 2019). With digital technologies systems becoming more complex, MBE should have ability to anticipate future trends and impacts of these changes and understand how it causes a paradigm shift in the maritime education sector. For instance, Peschl et al. (2020) argued that it is the responsibility of educators to identify the key competencies that are essential for learners in the future, and such ability is intertwined with pedagogy competency. In this context, MBE are required to understand the effects of social, economic and technological pressure of today’s market and assist learners in developing the competencies required.

*Strategic Planning* involves assessing the organizational, course and modular objectives and requirements to aid decision making in allocation of their own resources. Thai and Yeo (2015) concluded that logisticians should possess general business skills and knowledge including strategic planning. Furthermore, they have to work around deadlines by completing the syllabus in time for learners to sit for examinations (Hill and Chin, 2018). Apart from these considerations, strategic planning allows MBE to better locate the means and funds to acquire tools required to conduct lessons (Van Dam et al., 2010). Possessing strategic planning skills would enable MBE to develop strategic approaches that meet the objectives and requirements of the institutions (Kotler and Murphy, 1981). Therefore, strategic planning competency should be deemed essential for both educators and maritime business professionals to carry out tasks effectively.

*Transdisciplinary Thinkin*g refers to extent to which concepts across multiple disciplines are integrated to guide decisions. Transdisciplinary thinking entails the integration of multiple disciplines while taking into account of perspectives beyond these disciplines to provide a holistic approach (Klein, 1990; Choi and Pak, 2006). The maritime sector is interdisciplinary as it draws on knowledge from multiple disciplines and hands-on experiences (Levinson, 2016; Chen et al., 2018; Alfultis, 2019). Consequently, a high level of understanding of maritime concepts and the capacity to correlate materials from various disciplines is required for MBE to achieve learning objectives and prepare learners for the workforce.

*Global Mindset* refers to the ability to recognize different cultural values and cultivate global perspective in learners. The maritime industry is complex and multicultural (Progoulaki and Roe, 2011; Goodwin, 2020) that is a part of the global supply chain. Essentially, a global mindset drives educators to expand notions of learning beyond content integration (Goodwin, 2020). Further, understanding globalization is interrelated to pedagogy competency because it acts as a conceptual lens to reframe pedagogy, and is critical to successfully nurture learners who are intercultural empathetic.

### Pedagogy Competency

Pedagogy competency can be characterized by a person’s conscious activity designed to enhance learning in another (Mortimore, 1999). It encompasses the ability of educators to create a learning environment, evaluate behavioral aspects of learners and select appropriate strategies to achieve educational goals. MBE acts as a teacher, coach and mentor to drive learner development by continuously providing feedback and support. Some literature defines pedagogical competence as relying on subject deep knowledge and linking of theory and research to teach in a rational context (Ningtiyas, 2018). It implies a combination of pedagogical techniques that bring across learning content to meet the learning requirements established by higher education institutions. In a widely acknowledged study on pedagogy, Shulman (2005) identifies signature pedagogies as the ways of teaching beyond the purpose of understanding of the subject, but ‘preparation for accomplished and responsible practice in the service of others.’

*Classroom Management* refers to constructing a productive and stimulating learning environment. It entails physical arrangement of time, space and activities, and management of learners’ behaviors. Doyle (1980) suggested that a classroom is a complex environment that is subjected to immediacy, publicness, multidimensionality, unpredictability, history and simultaneity. Educators are expected to teach at a steady pace while maintaining clear direction during lessons (Guerriero, 2014). To do so in a complex, time-constrained environment, requires MBE to possess the ability to process a large amount of information rapidly and to sustain an orderly environment in the classroom.

*Learning Design* is the ability to formulate delivery approaches that engage learners and fulfill the learning outcomes. MBE are required to collect and analyze learner profiles and satisfy their learning needs (Mandinach and Gummer, 2013), and make decisions on the content to teach, the sequence and structure of the content, and how to best engage leaners. According to Bound and Chia (2020), learning design should incorporate principles such as authenticity, alignment, future-oriented and judgment. The authors also highlighted the importance of learner-centered teaching, which is in line with many researchers who identified the learning approach as “teaching and learning that emphasize student responsibility and activity in learning” (Newble and Cannon, 2013). Hence it is imperative for MBE to review the various teaching and learning approaches to best engage learners and improve their quality of teaching.

*Assessment Literacy* is the ability to gain clarity of purpose in assessment design and implement assessment methods and tools to evaluate learner progress. Acquiring a deeper understanding of purpose in assessment design is critical for educators to address learning gaps (Ministry of Education, 2020) because it allows them to gain a clearer grasp of what is necessary for learners. The past literature revealed three main criteria for assessment: assessment designs must be valid, fair and reliable; educators must maintain impartiality throughout the assessment implementation process; and learners must be clear of the assessment standards and requirements (Lloyd, 2013; SkillsFuture, 2020b). Satisfying the abovementioned requirements allow learners to conduct self-assessment of their work, spark enthusiasm and improve their motivation (Woytek, 2005).

*Learning Experience Evaluation* involves measuring the effectiveness of learning materials through learners’ feedback and performance. SkillsFuture (2020b) identifies learning experience evaluation as a tool that facilitates continuous improvement in the curriculum. Evaluation should be mutual whereby MBE address any performance issues and provide constructive feedback for learners to master the subject-matter. Besides providing opportunities to improve the curriculum and for learners to improve their understanding on the course content, Goe et al. (2008) found that monitoring learners’ progress contributes to educator effectiveness. Hence, acquiring learning experience skills would enhance the quality of teaching.

### Interpersonal Competency

Interpersonal competency and its effectiveness on students’ outcomes have been widely researched since the last decade (Reynolds, 1995). It is widely accepted that strong interpersonal skills are the hallmark for an educator (Reddy, n.d.; Zambas, 2018).

*Communication* is the ability to convey message clearly, concisely and effectively. It is generally agreed that communication is vital at all workplace to bring across messages and to prevent misunderstandings. Besides being a basis of competency requirement for maritime business professionals (Thai and Yeo, 2015; Chen et al., 2018), effective communication serves as a prerequisite for pedagogical practices. For instance, MBE need to present information such as the learning outcomes and learning content clearly and confidently and provide clear written and oral instructions in a manner that can be understood by learners (Serrano, 2019). Additionally, non-verbal communication skills such as listening and observing can be used as a tool to build trust (Blašková et al., 2014). Laar et al. (2020) found that establishing interpersonal relationships through effective communication is regarded as an essential skill in the 21st century. Hence, effective communication is crucial for MBE to establish interpersonal relationships with stakeholders and to improve educational quality.

*Leadership* is the ability to influence learners’ motivation through guidance and fostering of independent learning. Both educators and maritime business professionals require leadership skills to plan, lead, inspire and motivate others (Thai and Yeo, 2015; SkillsFuture, 2020b). In an educational context, learners’ motivation is underpinned by MBE’s capacity to self-motivate and motivate others, through instructor qualifications and own personality (Tang and Sampson, 2018). Some literature also suggest educators to lead by example and cultivate an open and collaborative learning culture, and prepare learners for leadership positions (SkillsFuture, 2020a). As MBE are directly involved in educating future maritime business graduates who are likely to hold managerial positions in the future, leadership competence should form as a requirement for them.

*Networking* involves a person’s capacity to identify and establish relationships with stakeholders (SkillsFuture, 2020a,b) such as students, faculty staff, professional bodies, industry professionals, schools and alumni. By establishing a professional network, educators are able to expand their influence beyond the classroom and acquire new teaching content and strategies. These could ensure the learning content are up to date which facilitate learners’ transition to full-time employment.

*Cognitive* refers to reasoning ability and general intelligence. Schmidt (2002) highlighted that cognitive capacities of individuals correspond with their performance at the workplace. Teaching as a profession is complex and demanding (Rotherham and Mead, 2003; Bardach and Klassen, 2020). Consequently, it is reasonable to assume that cognitive abilities are predictive of educators’ effectiveness. Some literature defined cognitive skills as the perception, attention, reaction times and reasoning skills (Chamorro-Premuzic and Furnham, 2010). Hence, possessing such abilities would enable MBE to react quickly to any challenging classroom situations.

*Social and Emotional* competence refers to the capacity to recognize own emotions and those of others, while managing these emotions effectively. Based on the Emotional Intelligence Domains and Associated Competencies model developed by Goleman et al. (2013), the four parts of emotional competence are: self-awareness, self-management, social awareness and relationship management. MBE are first and foremost teachers, who require self-awareness and social awareness to read students’ emotional currents and respond with empathy. Self-awareness is associated with effective teaching (Grant, 2017) and it encourages positive self-development of an individual (Sutton et al., 2015). Consequently, self-awareness would facilitate self-management, which is a critical domain in an era of continuous change. Relationship management encompasses the ability to influence learners, foster long-term developments of learners, and inspire learners to bring out the best in them (Media, 2017). Therefore, social and emotional competence should be deemed necessary for all educators to enhance teaching quality.

### Digital Competency

Digital competency can broadly be defined as the confident, critical and creative use of Information Communication Technologies to achieve work-related goals (Ferrari, 2012). Digitalization and automation will continue to play a significant role in the maritime industry. Studies have shown that in the next decade, maritime business professionals require skills of managing technology, including basic digital literacy and use of digital technologies and devices (Chen et al., 2018). The focus for MBE is not on technical skills, but rather how digital technologies could be used to enhance and innovate education and training.

*Technology Enabled Learning Delivery* involves leveraging of electronic media and latest digital technologies for lesson deliveries. On an international level, some ministries have promoted frameworks to support educators in experimenting with and adapting of digital technologies to suit learning objectives and deepen learning (Ministry of Education, 2020; Redecker, 2020; SkillsFuture, 2020b). Further, contemporary literature revealed the possibility of online education becoming the dominant mode of delivery in the future (Allen and Seaman, 2014; Boulougouris et al., 2019), increasing accessibility for learners (Simonson et al., 2019). Hence, technology-enabled learning delivery should serve as a basis to improve the quality of maritime business classes.

*Cyber Security* refers to basic understanding of measures and techniques to protect integrity of data. Cyber resiliency is required for all sectors in the maritime industry including ship and terminal operations, brokering, chartering and supply chain management (Alfultis, 2019). The transition to more integrated systems in the next decade require a focus on cyber security to prevent malicious attacks, and the responsibilities of helping learners to acquire cyber security acumen lies on higher level institutions (Fireman, 2018). To narrow the skills gap, MBE should possess basic understanding of encryption techniques, blockchain technologies and security control mechanisms (IMDA, 2020).

*Computational Thinking* is a reasoning skill to solve complex problems. It originates from computer science and could be distilled down to four elements: decomposition, pattern recognition, abstraction and algorithmic thinking (Wing, 2006). It is evident where a sense of urgency for joint efforts has been created for educational stakeholders to begin learning such skills (Looi, 2017). In the educational context, MBE are required to utilize computational models to guide decision making and develop skills (SkillsFuture, 2020b). Essentially, computational thinking brings about several advantages including enhancing of logical thinking and better articulation of problems. For instance, mastering algorithmic thinking allows MBE to create or use a well-defined series of steps to explain learning content (Sheldon, 2017). Mastering this set of competence enables integration of computational thinking into learning content which in turn facilitates educators’ lifelong learning process and enhances teaching quality.

### Maritime Foundational Competency

Maritime Foundational competency entails deep disciplinary knowledge that are cognitive and transferrable to learners. The academic research in maritime transport mainly focuses on logistics and supply chain professionals. According to Chen et al. (2018) and Sys and Vanelslander (2020), the maritime industry is a subset of the global logistics and supply chain. Hence, maritime foundational competency suggests the following five factors that would facilitate MBE’s dual role as a maritime business professional and educator: risk compliance and governance, maritime competency, supply chain competency, emerging maritime technology synthesis and sustainable developments.

*Risk Compliance and Governance* involves knowledge of laws and regulations of the maritime industry. Ziarati et al. (2010) argue that it is the role of educational stakeholders including educators and institutions to recognize the maritime industry practices and implement the curriculum program in accordance with the International Maritime Organization (IMO) model courses. The maritime industry is one of the most heavily regulated industries (ICS, 2020), and it is vital for every maritime business professional to gain knowledge of the compliance requirements and act in accordance with the industry best practices. Such knowledge has been identified in various supply chain skills frameworks (Murphy and Poist, 1991; Mangan and Christopher, 2005; Mageto and Luke, 2020). The skills and knowledge in this section are adapted from Thai and Yeo (2015) who have provided a wider variation of skills in this field including: risk management, commercial and transport law, maritime law, exporting and importing procedures, customs procedures and dangerous cargo regulations. Coupled with leadership qualities, MBE would be able to highlight and influence perception of students toward specific key themes of the industry (Boulougouris et al., 2019), such as cyber risk management in IMO (2021) mandate.

*Maritime* competency refers to acquiring deep understanding of concepts and industry practices in the maritime discipline. The concept of content knowledge originates as a part of a taxonomy of teacher knowledge by Shulman (1986), which has been widely recognized in literature (Ulferts, 2019). Chen et al. (2018) investigated the current and future employability skills for maritime business graduates from employers’ perspectives using a list of skills which were considered important by industry senior managers. Another academic research on the competency requirements of maritime workforce was conducted by Thai and Yeo (2015). Specifically, the authors advanced the business, logistics and management framework and further categorized the competencies into specialized fields such as generalist, maritime-related, and port-related. The maritime competencies in both studies are presented in Table 2.

**TABLE 2:** Maritime competencies.

Skills, knowledge and abilities		
(1) Shipping business operations and management	(10) Commercial law	(19) Cargo handling operations
(2) Overview of maritime industry	(11) Stevedoring operation	(20) Ship brokering and chartering
(3) International trade	(12) Marine insurance	(21) Maritime strategy
(4) Project management	(13) Freight forwarding	(22) Port strategic planning
(5) Port operations and management	(14) Systems concept	(23) Port productivity
(6) Transport systems (including intermodal transportation)	(15) Naval architecture	(24) Marketing
(7) Maritime geography	(16) Maritime economics	(25) Ship routing and scheduling
(8) Financial management	(17) Port safety and security	
(9) Financial accounting	(18) Fleet size and mix decisions	

*Compiled by the authors.*

*Supply Chain* competency refers to the understanding of concepts and industry practices in the supply chain discipline. It is crucial for MBE to be equipped with supply chain skills as manufacturing and distribution practices have an impact on the maritime industry. Supply chain skills have been discussed under various frameworks, with little agreement among researchers on the type of skills, categories and their terminology (Tatham et al., 2017). Further, in view of the changing environment, researchers have advanced the existing frameworks. For instance, Mageto and Luke (2020) identified supply chain management skills such as: demand forecasting inventory management, warehousing, procurement and 3PL collaborations.

*Emerging Maritime Technology Synthesis* involves identifying new technologies in the maritime industry to support learning materials. The use of information and communications technology applications is a future trend in maritime industry (Chen et al., 2018). Monitoring and integrating new and emerging technological products of the industry into learning materials (SkillsFuture, 2020b) enable MBE to stay relevant about the trends and developments. Consequently, maritime business graduates are exposed to the digital technologies used in the industry which would enhance their digital readiness before entering the workforce.

*Sustainable Development* refers to understanding sustainable concepts and developments in the maritime and logistics industry. There is a rising awareness of environmental consequences as a result of intensified international trade and shipping (Song and Panayides, 2012). Therefore, MBE need to be familiar with concepts such as corporate social responsibility, sustainable shipping and green logistics. Sustainable shipping manages the sustainable development of the maritime sector, characterized by threefold classification: the society, environment and economy (EMSA, 2020). The importance of sustainable shipping for MBE is supported by the framework developed for sea transport professionals (SkillsFuture, 2020a). Similarly, green logistics consist of environmental assessment, reverse logistics and green supply chains (Abukhader and Jönson, 2004). In order to meet the rising demand for sustainable efforts in the maritime industry, MBE should familiarize themselves with these concepts.

## Methodology

The conceptualization of a competency framework for MBE involved a systematic review of the competency requirements for both general educators and onshore maritime professionals including maritime business graduates and supply chain professionals. This section explains how articles were searched and subsequently, how the questionnaire was developed.

### Design of Review

The articles chosen for this study were searched through Scopus, for two reasons. Firstly, it is the most recognized bibliographic database available for electronic academic literature search (Falagas et al., 2008; Pinho and Mendes, 2017). Secondly, it is managed by the largest publishing house, Elsevier, thus demonstrating good coverage of publications. The search was conducted through three layers of keywords (Table 3). In addition, the asterisk wildcard is very useful to find topics that come in plural and singular forms. For that reason, it was used to pick up variations of the keywords (e.g., searching [competen*] returned “competent,” “competency,” “competencies,” etc.). In order to obtain articles that are relevant to this study, the search was limited to subject areas on “engineering,” “social sciences,” “business, management, and accounting,” “multidisciplinary,” “environmental science,” and “psychology.”

**TABLE 3:** Procedure and results of literature extraction.

Search Keywords	Search Results
(a) First-layer search structure	467,377
Competen* OR skill*	
(b) Second-layer search structure	2,599
Competen* OR skill*	
AND	
Maritime OR ship* OR supply chain OR logistic*	
(c) Third-layer search structure	423
Competen* OR skill*	
AND	
Maritime OR ship* OR supply chain OR logistic*	
AND	
Educ* OR teach* OR lectur* OR pedag*	

Two layers of search were conducted. The first layer of search was related to competency requirements, which included keywords such as “competency” and “skill.” The search was further narrowed through a second layer of keywords that are related to the context. Keywords such as “maritime,” “maritime business,” and “shipping” were used to filter out articles that are related to the maritime industry. Since MBE play a dual role as both maritime professional and an educator, the third layer of search was related to the education aspect and included keywords including “educator,” “teacher,” “lecturer,” and “pedagogy.” The search terms yielded 423 articles, and the titles and abstracts were examined to extract potentially relevant articles. Of these, 365 articles were excluded as they were not related to educators, maritime, logistics and supply chain competencies. Fifty-eight articles were selected for full-text reading with the sub-competencies identified. Subsequently, the predominant themes of the sub-competencies were qualitatively grouped into key dimensions. A comprehensive list of competencies identified from the systematic review were included in the questionnaire to test their significance to MBE’s teaching competency. See Table 1.

### Scale Development

The survey questionnaire comprises two sections. The first section aims to identify the importance of the five main competencies and the respective 22 subscales presented in Table 1. The terminology of each competency was also defined. Respondents were asked to rate each competency on the Likert scale with 1 indicating “Strongly Disagree” and 9 as “Strongly Agree” whether it is critical to MBE. The second section of the questionnaire captures demographic information on the respondents and their perceived teaching performance. Perceived teaching performance was measured using four variables adapted from scales presented in Kaldi (2009). The scales, though originally targeted at directly pre-service educators, covered content that were seen as being directly relevant to educators’ perceived teaching performance:

*I feel competent about preparing teaching plan before carrying out teaching duties.*

*I feel competent about carrying out teaching.*

*I feel competent about creating a pleasant classroom climate.*

*I feel competent in using various digital means and materials for my teaching.*

### Survey Design and Administration

All Educators teaching in all maritime business-related disciplines within the top ten schools were included in the targeted sample population. They were selected Based on the Top 30 Worldwide Maritime School Rankings listed by The Hong Kong Polytechnic University (2015). In addition, the sample frame includes faculty members from the World Maritime University. The university is founded by the United Nations’ specialized agency, International Maritime Organization, and plays a significant role in the maritime and ocean education (IMO, 2019). One may expect that MBE who had been employed by the top maritime universities would be well positioned to reflect the competency requirements that are influential in their development as educators. Accordingly, a total of 196 contacts were obtained from the databases of the respective universities’ websites.

An invitation e-mail was sent to all potential participants to request for their participation in the survey. Those who accepted the invitation were directed to a website for their completion. Subsequently, two reminders were sent to the participants who had not completed the survey. Data collection was performed over a period of 2 months.

### Demographic Profile

Out of 196 invitation e-mail sent to the potential participants, 116 completed all questions on the survey, with a completion rate of approximately 58.6%. The demographic characteristics of respondents are summarized in Table 4. As evident in Table 4, the responses show a good variety in terms of years of experience, with participant groups fairly spread across the periods of not more than 5, 6–10, 11–15 and over 16 years. Majority of the respondents hold senior lecturer or lecturer position (41.4%) and specialize in maritime economics (48.3%).

**TABLE 4:** Demographic characteristics of respondents (*n* = 116).

Demographic information	No. of respondents	*n* (%)
***Years of experience in maritime business education***		
<5 years	18	15.5
6–10 years	34	29.3
11–15 years	36	31.0
Over 16 years	28	24.1
***Job position***		
Senior lecturer/lecturer	48	41.4
Assistant professor	28	24.1
Associate professor	26	22.4
Professor and above	14	12.1
***Maritime specialization***		
Maritime business management	40	34.5
Maritime law	10	8.6
Maritime economics	56	48.3
Maritime technology	10	8.6

### Data Analysis Method

Using the collected data, this study conducts an exploratory factor analysis on the competency measurement items to extract the underlying latent factors (i.e., competency dimensions). The purpose is to determine whether the measurement items are classified in the same latent factors as posited by this study. In addition, the reliability and validity of the latent factors are also assessed using Cronbach’s alpha and cross-loading analysis. Thereafter, hierarchical regression analysis is conducted to examine the effects of the competency dimensions on teaching performance.

## Results and Discussion

### Exploratory Factor Analysis

Exploratory factor analysis is a statistical technique primarily used to reduce multiple dimensions into a smaller set of dimensions and establish underlying relationships between scales and dimensions (Williams et al., 2010). It is commonly applied when there are no expectations of the number or nature of dimensions. As a rule of thumb, the minimum sample size is 100 in order for the analysis results to be considered reliable (Hair, 2009). Exploratory factor analysis was employed using principal component analysis as the extraction method.

**FIGURE 2:**
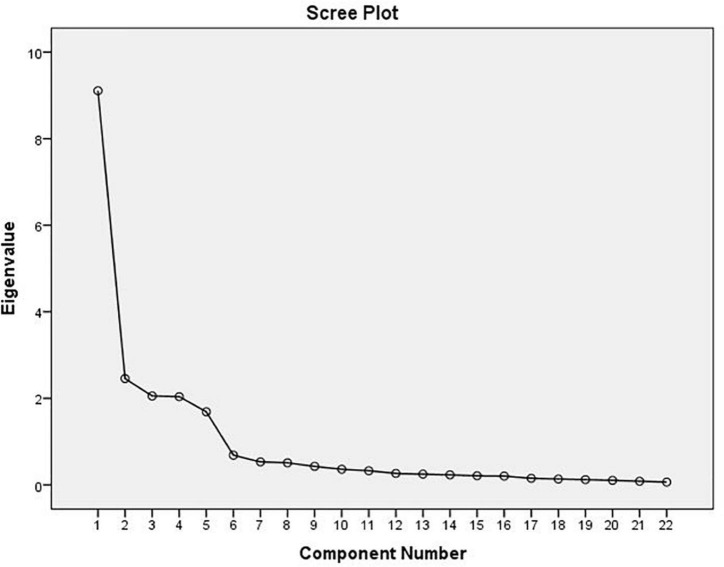
Scree plot.

As illustrated in Figure 2, Cattell (1966)’s scree test indicated five factors that were above the point of inflexion. This was supported by the Kaiser–Meyer–Olkin criterion (eigenvalue > 1), where the initial factor extraction suggested a five-factor solution to represent the 22 items.

Under the assumption that the factors are correlated (Yong and Pearce, 2013), direct oblimin rotation was applied to condense all 22 measurement items into a reduced set of factors that explain the underlying structure of the competency requirements. The competency requirements were sorted in accordance with the factor that they are most correlated with (i.e., highest eigenvalue), as presented in Table 5.

**TABLE 5:** Exploratory factor analysis – rotated component matrix.

Competency requirements	Business	Maritime	Interpersonal	Digital	Pedagogy
Innovation	0.835	–	–	–	–
Environmental Analysis	0.783	–	–	–	–
Strategic Planning	0.894	–	–	–	–
Transdisciplinary Thinking	0.831	–	–	–	–
Global Mindset	0.619	0.526	–	–	–
Risk Compliance & Governance	–	0.774	–	–	–
Maritime–specific	–	0.767	–	–	–
Supply chain–specific	–	0.681	–	–	–
Emerging Technology Synthesis	–	0.845	–	–	–
Sustainable Development	–	0.836	–	–	–
Communication	–	–	0.813	–	–
Leadership	–	–	0.817	–	–
Networking	–	–	0.855	–	–
Cognitive	–	–	0.834	–	–
Social and Emotional	–	–	0.860	–	–
Technology Enabled Learning Delivery	–	–	–	0.834	–
Cyber Security	–	–	–	0.829	–
Computational Thinking	–	–	–	0.889	–
Classroom Management	–	–	–	–	0.820
Learning Design	–	–	–	–	0.853
Assessment Literacy	–	–	–	–	0.804
Learning Experience Evaluation	–	–	–	–	0.853

Eigenvalue (λ)	9.107	2.456	2.054	2.039	1.688
Total variance explained (%)	41.396	11.165	9.338	9.267	7.674
Cumulative percentage variance (%)	41.396	52.561	61.889	71.166	78.840

Unidimensionality refers to the homogeneity of the items that measure a single latent factor. Specifically, unidimensionality is ascertained when each key competency requirement only represents a single concept [i.e., each item exhibits a high factor loading (eigenvalue) on a single factor and minimum cross-loading with other factors is observed]. As presented in Table 5, the eigenvalue (λ) > 1 verified that each factor accounted for the variance of more than one measurement item, which supports the parsimony principle when conducting exploratory factor analysis. Each measurement item recorded factor loadings above 0.60 value. Maskey et al. (2018) recommended suppressing factor loadings with values less than 0.4 because loadings above 0.4 are considered vital for discussion. Following this recommendation, factor loadings below 0.40 were suppressed, revealing one cross-loading item, i.e., “global mindset.” This measurement item recorded factor loadings of 0.619 and 0.526 for the business and maritime factors, respectively. To reiterate, global mindset is a 21st century competency that is defined as the capacity to “function effectively within environments characterized by high cultural and business complexity” (Andresen and Bergdolt, 2017). As a result of globalization, educating global mindset and cultural intelligence skillsets have been widely discussed by academics (Ghosh, 2019; Goodwin, 2020). Further, it is deemed as a prerequisite for successful functioning in the ever-changing global business landscape and complements other competency dimensions. For instance, global mindset allows MBE to gain knowledge and understanding of the global business, cognitive complexity and cultural acumen (Cseh et al., 2013). In the maritime context, gaining a global perspective allows MBE to examine the contemporary phenomenon, such as geopolitics, power dynamics and economic impacts on countries, and how these global events affect the development of the maritime industry. In the pedagogy element, MBE would be able to prepare curriculum from a globalization stance, incorporating international and intercultural elements (Knight, 2017). Global mindset also improves interpersonal skills and is reflected in cosmopolitanism (e.g., respect for cultures, openness and sensitivity) and participation of social networks. Gaining such perspective prepares MBE for diversity and allowing them to be culturally empathetic. Therefore, “global mindset” is viewed as a vital competency required by MBE and has been retained in the framework. All items were retained for further analysis because they have high loadings on the same factors (i.e., >0.6).

Internal Consistency Reliability was evaluated using Cronbach’s alpha coefficient with alpha value above 0.70 being considered acceptable (Nunnally, 1978). A summary of the mean, standard deviation, correlation and Cronbach alpha values of each key competency requirement is presented in Table 6. The factors are proven to be reliable as the alpha values are high, 0.93 for Business, 0.89 for Maritime, 0.90 for Pedagogy, 0.83 for Digital and 0.94 for Interpersonal, respectively. In this study, no variable is dropped as the alpha values of the competency requirements exceeded the cutoff value.

**TABLE 6:** Descriptive statistics, reliability and Pearson’s correlations.

Competency requirement	Mean	Standard deviation	Correlations				
			
			Business	Maritime	Pedagogy	Digital	Interpersonal
Business	4.55	2.08	(0.93)				
Maritime	5.89	1.91	0.59	(0.89)			
Pedagogy	4.55	2.19	0.58	0.53	(0.90)		
Digital	4.96	1.53	0.30	0.40	0.28	(0.83)	
Interpersonal	5.97	1.58	0.46	0.42	0.39	0.38	(0.94)

*n = 116.*

*Cronbach’s α reliabilities for the scales are shown along the diagonal.*

The analysis on both unidimensionality and reliability indicate that each competency requirement is representative of its measurement items, and thus supports the subsequent regression analysis.

### Interpretation of Factors

With reference to Table 5, the five-factor structure in this study composing 22 items collectively account for approximately 78% of the total measurement variance, which is considered acceptable in the field of social sciences (Zikmund et al., 2013). Business Competency is reflected by the first five measurement items. Its total variance of 41.4% reveals this dimension as a dominant, first factor. It involves business acumen through attributes such as innovation, environmental analysis, strategic planning, transdisciplinary thinking and global mindset.

Maritime Competency is collectively represented by the second group of measurement items. It relates to content knowledge that is deemed necessary to ensure teaching quality and assist learners to understand subject-matter theories. This factor includes risk compliance and governance, maritime-specific, supply-chain specific, emerging technology synthesis and sustainable development.

Interpersonal Competency comprises the next five measurement items. The items primarily involve human interaction and address the ability of MBE to provide support for learners’ wellbeing. The factors include communication, leadership, networking, cognitive and social and emotional aspects of MBE which are necessary to keep learners actively engaged during lessons.

The fourth factor, Pedagogy Competency, is characterized by four measurement items. These items imply a combination of pedagogical techniques to successfully bring across learning content through classroom management, learning design, assessment literacy and learning experience evaluation.

Digital Competency represents the last group of measurement items. This factor is viewed as an emerging skillset in response to the rise of digitalization. It consists of technology-enabled learning delivery, cyber security and computational thinking.

### Hierarchical Regression Analysis

On the basis of examining the effects of the five key competency requirements on perceived teaching performance, a hierarchical regression was conducted. Teaching performance was regressed on the predictors in a sequential manner with all control variables being analyzed, followed by the five key competency requirements. The hierarchical regression analysis results predicting the effects on teaching performance are presented in Table 7.

**TABLE 7:** Hierarchical regression analysis with perceived teaching performance as dependent variable.

	Model 1 Controls		Model 2 Competency requirements	
		
Variables (i)	β	Sig.	β	Sig.
***Control variables***				
Years of experience	0.143	0.134	0.114	0.056
Job position	0.032	0.733	<0.001	0.993
***Competency requirements***				
Business			0.162	0.043
Maritime			0.193	0.015
Pedagogy			0.379	<0.001
Digital			0.131	0.048
Interpersonal			0.176	0.011
***Model’s summary statistics***				
*n*	116		116	
Δ*F*	1.357		38.358	
*Sig. of*Δ*F*	0.262		<0.001	
*R*^2^	0.023		0.648	
Δ*R*^2^	0.023		0.625	

Two regression models were analyzed. In Model 1, teaching performance was regressed on the demographical aspects of MBE including “years of experience” and “job position” which are the control variables. The model is found to be insignificant (*F* = 1.357, *p* > 0.05) which indicates that teaching profiles of educators have negligible effects on teaching performance. Job positions (β = 0.032, *p* > 0.05) are awarded based on several influential aspects such as research excellence or teaching excellence (Aylett and Gregory, 2012). Further, Dunkin (1990) found that educators perceive institutions to place higher priority in research and publication for their own career advancements. Given that career advancements could be attributed by several factors, it could be assumed that an educator with a “professor” title does not necessarily possess greater teaching performance than a “lecturer.” Similarly, the years of experience (β = 0.143, *p* > 0.05) have no significant effect on teaching performance. In a widely acknowledged study conducted by Klassen and Chiu (2010), the authors found that years of experience has non-monotonic relationships with educators’ self-efficacies. Educators’ self-efficacies may vary over the course of a career, and those at mid-to-late career stages may cut down overambitious goals due to ebbing self-efficacy (Bandura, 1997). On the other hand, educators without prior employment in university or those on probation are more willing to help learners (Dunkin, 1990). The willingness could be a result of intrinsic motivation and enthusiasm. Nevertheless, an educators’ skills, knowledge and effectiveness may change over time and requires a lifelong process of development in maintaining them. This also indicates that veteran educators may not possess greater level of teaching performance than novice educators, especially if they do not constantly reflect on their personal attributes and environmental circumstances to seek self-improvements.

In addition to the control variables in Model 1, five competency requirements were included in the subsequent hierarchical regression model (i.e., Model 2). The purpose of Model 2 is to identify the main linear effects of the identified competency dimensions on teaching performance. Model 2 is significantly better than Model 1 (Δ*F* = 38.358, *p* < 0.05), and accounts for 64.8% (*R*^2^) of the variance in teaching performance, which is much higher than that in the case of Model 1 (2.3%). The hierarchical regression confirms that teaching performance can be predicted by the five competency requirements. The control variables in Model 2 remain insignificant, which was in line with the argument that years of teaching and position do not have a significant contribution to teaching performance (Zhu et al., 2013). All five competency requirements have significant effects on teaching performance, which indicates that MBE feel more competent in teaching when they possess business acumen, in-depth subject knowledge, pedagogy knowledge, interpersonal skills, and the ability to utilize educational technology.

Amongst the five competency requirements, pedagogy (β = 0.379, *p* < 0.05) showed the strongest, positive effect on teaching performance. The result is unsurprising as pedagogy serves as the backbone of teaching performance. For instance, gaining the capacity to analyze learners’ demographics would enable MBE to make hypothetical connections to their preferred learning styles and formulate teaching plan that is best suited for them. The competence in carrying out teaching also lies in the ability to evaluate learner progress, address learning gaps and offer the opportunity for learners to conduct self-assessment. These aspects could be covered when MBE are transparent about the assessment rubrics (SkillsFuture, 2020b) and provide continuous feedbacks which contribute to educator effectiveness (Goe et al., 2008). However, it is noteworthy that the role as an educator has a greater significance in comparison to the role as maritime business professional for MBE. Besides highlighting the importance of pedagogy competency, this analysis also reveals the increasing need for MBE to review the various teaching and learning approaches to best engage learners and improve their quality of teaching.

Both maritime foundational competency (β = 0.193, *p* < 0.05) and interpersonal competency (β = 0.176, *p* < 0.05) exert relatively similar, positive effects on teaching performance. Acquiring deep disciplinary knowledge is deemed as a pre-requisite for an educator to ensure teaching quality (Shulman, 2005). Although possibly self-evident, possessing sound disciplinary knowledge of subject content is required in order to teach it. For example, acquiring in-depth content knowledge would instill a greater sense of confidence in MBE when conducting lessons as they are able to draw on the subject knowledge and assist learners to understand the theories. In addition, MBE should acquire excellent interpersonal skills to facilitate knowledge transfer to learners and to create a positive classroom climate. The classroom climate is fundamentally interpersonal in nature (Barr, 2016), and building a strong rapport with learners is essential in the development of positive classroom environment. Therefore, MBE that engage in interpersonal behaviors, such as demonstrating empathy, active listening and communicating concern for learners’ academic progress or personal matters, would create a sense of belonging for learners. This finding corroborates previous research that has suggested subject-matter knowledge and social competency as the core aspects of an educator (Shulman, 2005; Zhu et al., 2013).

This study also found that business competency (β = 0.162, *p* < 0.05) was linked with perceived teaching performance. In fact, business competency is positively associated with pedagogy, whereby MBE’s ability to adopt fresh perspectives would facilitate learning design formulation and preparation of teaching plan. This is aligned with Fraser and Treagust (1986) who have found educator’s innovation to be an essential aspect of higher education classroom climate. Besides possessing creative thinking, which is reflected in educators’ teaching styles and methods, other business skills such as environmental analysis enable MBE to be forward-looking and identify trends in the maritime sector. MBE’s understanding of how these changes are causing a paradigm shift in the maritime education sector could be reflected in curriculum redesign to ensure that the teaching content are up-to-date.

Though digital competency is the lowest significance component to enhance teaching performance (β = 0.048, *p* < 0.05), it is recognized as an essential and adaptive competency to support teaching and learning (Huda et al., 2017). Digital competency addresses the perceived competence of MBE in using digital means and electronic materials for teaching. Competence in this element requires MBE to understand what to best teach with, and through digital means in order to enhance subject learning outcomes. In a diverse and digitally mediated environment, MBE need to recognize the benefits of information technology and tap on the opportunities that arise as a result of such changes. For instance, digital technologies could be used to enhance teaching methods. As suggested by Koehler and Mishra (2009) and Falloon (2020), pedagogical and learning design competence, content knowledge competence and technological competence are three core aspects that integrate to establish a solid foundation for educators to make informed decisions about use of digital tools and to improve their teaching effectiveness. Hence, digital competence is viewed as a complementary competence to enhance teaching quality.

This study reveals that the five competency requirements do not hold equal significance in teaching performance. Specifically, MBE should prioritize pedagogy competency over maritime foundational knowledge and interpersonal skills. Therefore, pedagogical elements such as classroom management, learning design, assessment literacy and learning experience evaluation should be the main focus of MBE in order to improve their teaching performance.

## Conclusion

### Main Findings

In a digitally mediated environment where information is accessible to everyone, the knowledge and skills acquired by MBE are evolving rapidly, and may become outdated by the time the learners enter the workforce (Alop, 2019; Boulougouris et al., 2019). Recognizing the rising importance of acquiring relevant and up-to-date competencies for MBE, this study shed light on the contemporary competency requirements and the effects of these competency dimensions on MBE teaching performance. Based on a systematic review of the contemporary literature, this study proposed a conceptual framework consisting of five key competency requirements and 22 sub-competencies that can improve teaching performance. A survey questionnaire was designed and administered to the 196 faculty members of the leading maritime universities. Subsequently, the exploratory factor analysis and hierarchical regression analysis was adopted to identify the main dimensions of the MBE framework, and to examine the effects of the competency dimensions on teaching performance. This study revealed, in descending order of their importance to teaching performance, (1) pedagogy, (2) maritime foundational, (3) interpersonal, (4) business, and (5) digital competency, along with 22 sub-competencies as essential aspects of MBE.

This study revealed pedagogy competency to be the most important dimension influencing teaching performance, which indicates that the role as an educator takes precedence over the role as maritime business professional. Specifically, it is the responsibility of MBE to transfer knowledge of the subject content to learners and to train them to think as professionals in maritime discipline. Besides being an essential factor for high-quality teaching, good pedagogical skills improve learners’ outcome and promote greater interest in the subjects taught (Ulferts, 2019). Hence, understanding the fundamentals of pedagogy is an overarching skill for MBE to enhance learners’ chances of success.

Parallel to the above finding is the importance of pedagogical skills such as classroom management, learning design, assessment literacy and learning experience evaluation for MBE to improve their teaching performance. It also revealed that the required competencies for MBE should go beyond the three main aspects of general educators, namely, pedagogy, maritime and digital (Shulman, 2005), and encompass interpersonal and business competencies. Therefore, besides acquiring subject-related knowledge, MBE are required to possess excellent interpersonal competence to engage learners during lessons and to provide support for their mental wellbeing. Empathy and emotional intelligence of educators are necessary to create a sense of belonging for learners which is associated with learners’ self-efficacy and intrinsic motivation (Freeman et al., 2007). For instance, Lavy and Naama-Ghanayim (2020) found that learners’ perceptions of educators caring for them has a positive impact on their own well-being, motivation, self-esteem and school engagement. Thus, interpersonal competency of MBE is crucial to prepare graduates in terms of knowledge and mental health. In addition, it was found that the proposed conceptual framework would contribute to teaching performance of MBE.

### Implications of Study

This research has both theoretical and practical implications. Despite the extant literature in maritime education, research on MBE is scant, and no research has explored the competency requirements of MBE. Currently, the competency standards only apply for seafarers under the STCW 1978/1995 convention and maritime business professionals such as container shipping logisticians and maritime business graduates. Considering the vital role of MBE in facilitating knowledge transfer and delivering industry-ready graduates in changing industrial landscapes, it is crucial that the competency profile of MBE is developed to establish competency standards in this important part of the chain. Therefore, this proposed competency framework serves as a reference point for future research in competency requirements that are critical for MBE to achieve greater teaching performance. The higher education classroom is a multidimensional environment (Barr, 2016), and MBE need to recognize that the competency requirements are uniquely intertwined and characterized by a complementary relationship in which they should not be considered in isolation. For instance, environmental scanning, or establishing of professional networks with alumni and industry professionals, would help MBE to expand their influence beyond the classroom. By learning about the latest developments in the industry, they would be able to identify emerging skills required by maritime business graduates, which could be reflected in pedagogical approaches such as changes in curriculum or teaching styles. These could ensure the learning content are up-to-date which facilitate learners’ transition to full-time employment. Additionally, the findings of this study may be useful for pre-service and practicing MBE by providing the opportunity to conduct self-assessment and to align with the industry standards. The five identified competency requirements serve as “aim points” toward which MBE could use to evolve their practice. This facilitates lifelong learning, as it is crucial for them to deepen their skills to meet existing and emerging demands of the maritime sector (SkillsFuture, 2020a). It is also noteworthy that the success of meeting these requirements relies on MBE capacities for flexibility, willingness to adapt and readiness to explore how these dimensions interrelate to support effective teaching.

### Limitations and Future Research

As the scope of the current study encompasses views of MBE in the leading maritime universities, it is expected that the views may not represent all MBE. Therefore, it is proposed that this research can be extended further by involving more maritime institutions, specifically those that offer maritime business programs to participate and validate the results of this research. An interesting area for future research would be the design of such trainings and at various levels of their career path. Additionally, future research studying the effects of the educators’ age, gender and previous work experiences on the core competencies would be meaningful in shedding light on competency requirements that different profiles of educators should note.

## Data Availability Statement

The original contributions presented in the study are included in the article/supplementary material, further inquiries can be directed to the corresponding author.

## Ethics Statement

The studies involving human participants were reviewed and approved by Nanyang Technological University – Institutional Review Board (NTU-IRB). The patients/participants provided their written informed consent to participate in this study.

## Author Contributions

LT and KY: conceptualization, methodology, and visualization. LT, KY, and HL: writing—original draft preparation, and writing—review and editing. KY: funding acquisition. All authors read and agreed to the published version of the manuscript.

## Conflict of Interest

The authors declare that the research was conducted in the absence of any commercial or financial relationships that could be construed as a potential conflict of interest.

## Publisher’s Note

All claims expressed in this article are solely those of the authors and do not necessarily represent those of their affiliated organizations, or those of the publisher, the editors and the reviewers. Any product that may be evaluated in this article, or claim that may be made by its manufacturer, is not guaranteed or endorsed by the publisher.
